# Modulation of Purkinje cell complex spike waveform by synchrony levels in the olivocerebellar system

**DOI:** 10.3389/fnsys.2014.00210

**Published:** 2014-10-30

**Authors:** Eric J. Lang, Tianyu Tang, Colleen Y. Suh, Jianqiang Xiao, Yuriy Kotsurovskyy, Timothy A. Blenkinsop, Sarah P. Marshall, Izumi Sugihara

**Affiliations:** ^1^Department of Neuroscience and Physiology, New York University School of MedicineNew York, NY, USA; ^2^Department of Systems Neurophysiology, Graduate School of Medical and Dental Sciences, Tokyo Medical and Dental UniversityTokyo, Japan; ^3^Center for Brain Integration Research, Tokyo Medical and Dental UniversityTokyo, Japan

**Keywords:** inferior olive, complex spike, zebrin, synchrony, spikelets, gap junctions

## Abstract

Purkinje cells (PCs) generate complex spikes (CSs) when activated by the olivocerebellar system. Unlike most spikes, the CS waveform is highly variable, with the number, amplitude, and timing of the spikelets that comprise it varying with each occurrence. This variability suggests that CS waveform could be an important control parameter of olivocerebellar activity. The origin of this variation is not well known. Thus, we obtained extracellular recordings of CSs to investigate the possibility that the electrical coupling state of the inferior olive (IO) affects the CS waveform. Using multielectrode recordings from arrays of PCs we showed that the variance in the recording signal during the period when the spikelets occur is correlated with CS synchrony levels in local groups of PCs. The correlation was demonstrated under both ketamine and urethane, indicating that it is robust. Moreover, climbing fiber reflex evoked CSs showed an analogous positive correlation between spikelet-related variance and the number of cells that responded to a stimulus. Intra-IO injections of GABA-A receptor antagonists or the gap junction blocker carbenoxolone produced correlated changes in the variance and synchrony levels, indicating the presence of a causal relationship. Control experiments showed that changes in variance with synchrony were primarily due to changes in the CS waveform, as opposed to changes in the strength of field potentials from surrounding cells. Direct counts of spikelets showed that their number increased with synchronization of CS activity. In sum, these results provide evidence of a causal link between two of the distinguishing characteristics of the olivocerebellar system, its ability to generate synchronous activity and the waveform of the CS.

## Introduction

The Purkinje cell (PC) of the cerebellum displays two types of action potentials: simple spikes, which are generated intrinsically and in response to excitation by the mossy fiber/parallel fiber system (Llinas and Sugimori, 1980; Häusser and Clark, 1997; Raman and Bean, 1999; Cerminara and Rawson, 2004), and complex spikes (CSs), which are evoked by the olivocerebellar system (Eccles et al., 1966a). Unlike simple spikes, which are essentially standard action potentials, the CS has a distinctive and variable waveform consisting of an initial spike followed by small spikelets that vary in number and amplitude. This distinctive waveform has led to hypotheses about the function of CS activity, and the spikelets, in particular, have been postulated to be an important functional parameter of CS activity. For example, the number of spikelets in a CS has been hypothesized to be a readout of the state of the PC at the time of the CS (Eccles et al., 1966a, 1967), and has been correlated with the type and strength of synaptic plasticity (LTD or LTP) induced by climbing fiber activity (Mathy et al., 2009).

What makes the CS waveform a particularly attractive possibility for being a functional parameter of olivocerebellar activity is that it is variable, and thus potentially subject to modulation. However, the causes underlying this variation have received relatively little attention, perhaps because the CS has often been incorrectly thought of as an all-or-none event (reviewed in Najafi and Medina, 2013) when it is probably best conceived of as a composite of many all-or-none events (Llinas and Nicholson, 1971).

Potential causes of CS waveform variation can be broadly split into those related to the state of the cerebellar cortex, the PC in particular, and those related to the state of the inferior olive (IO). Although this division is almost certainly not absolute, because of the closed loop nature of the circuits connecting the IO and the cerebellum (Ruigrok, 1997; Marshall and Lang, 2009; Chaumont et al., 2013), it provides a useful experimental and conceptual framework. For example, evidence that cerebellar cortical activity can modulate the CS waveform includes classic results, such as that when a CS is conditioned by the activation of molecular layer interneurons it has a reduced number of spikelets (Eccles et al., 1966b), and more recent ones, such as the demonstration of a negative correlation between glutamate transporter EAAT4 expression levels in PCs and spikelet number under *in vitro* conditions (Paukert et al., 2010).

The possibility that the state of the IO plays a significant role in determining the CS waveform is raised by the ability of IO neurons to discharge high frequency bursts of spikes rather than individual action potentials (Armstrong and Harvey, 1966, 1968; Crill, 1970), and by the fact that the size of these bursts is correlated with number of spikelets in the resulting CS (Mathy et al., 2009). Thus, factors that modulate the size of the IO bursts would likely also modify the CS waveform; and indeed, several such factors relating to the subthreshold oscillation displayed by IO neurons, including its amplitude, have been identified experimentally (Maruta et al., 2007; Mathy et al., 2009; Bazzigaluppi et al., 2012; De Gruijl et al., 2012). Moreover, modeling results predict that the amplitude of the subthreshold oscillation should be inversely related to the degree of electrical coupling between IO neurons (De Gruijl et al., 2012), suggesting that the latter should also influence the CS waveform.

Here we used multiple electrode recording of CS activity to investigate whether the CS waveform is in fact affected by the state of coupling among IO neurons. These recordings allowed us not only to measure the CS waveform, but also to monitor the state of the IO, because of the one-to-one relationship between CSs and IO discharges, and the fact that synchronous CS activity reflects the effective electrical coupling pattern among IO neurons (Lang et al., 1996; Lang, 2001, 2002; Long et al., 2002; Blenkinsop and Lang, 2006; Marshall et al., 2007; Onizuka et al., 2013). The present results indicate that the level of CS synchrony is causally linked to the CS waveform, and thus provide evidence that the state of electrical coupling among IO neurons is a mechanism by which this waveform may be modulated.

## Methods

Experiments were performed in accordance with the NIH's *Guide for the Care and Use of Laboratory Animals*. Experimental protocols were approved by the Institutional Animal Care and Use Committee of New York University School of Medicine.

### General surgical and recording procedures

In most experiments, female Sprague-Dawley rats (225–300 g) were initially anesthetized with ketamine (100 mg/kg) and xylazine (8 mg/kg) intraperitoneally. Supplemental anesthetic was given via a femoral catheter to maintain a constant depth of anesthesia. In some experiments, urethane was used as the anesthetic with an initial dose of 1.5 g/kg followed by supplemental doses of 0.3 g/kg as needed, all given intraperitoneally. In all experiments, the depth of anesthesia was assessed by a paw pinch and the absence of spontaneous movements. Rectal temperature was maintained at 37°C using a heating pad connected to a temperature control system. To gain access to the cerebellum, animals were placed in a stereotaxic frame, and a craniotomy was performed to expose the posterior lobe of the cerebellum. The dura mater was then removed, and the cortical surface was stabilized and protected by covering it with a platform constructed from an electron microscope grid that was pre-embedded in a thin sheet of silicone rubber and supported by tungsten rods. The platform was cemented to the skull of the animal. For further details on the platform construction, see Sasaki et al. (1989).

Extracellular recordings of CS activity were made using single and multiple electrode techniques. In both cases, recording electrodes were implanted by driving them through the rubber and into the apex of the folium using a micromanipulator. Most recordings were from crus IIa except where specified in the Results, in which cases recordings were from vermis lobule VIII.

Single electrode recordings were made with glass micropipettes containing 2 M NaCl solution and were used to obtain high signal-to-noise recordings of CS activity, usually at the PC somatic level (typically 250–300 μm below the cortical surface). The presence of simple spikes and the initial positivity of the CS waveform were used as indicators that these recordings were made at, or close to, the PC soma.

A multiple electrode technique was used to obtain recordings from arrays of PCs simultaneously. In this case, electrodes were typically implanted 75–150 μm below the surface. At these depths simple spike activity is not observed, and thus CSs can be easily recorded in isolation from the PC dendrites. For these recordings the electrode solution was a 50/50 mixture of 2 M NaCl solution and glycerol. Electrodes were implanted sequentially, with each electrode being released from the manipulator upon isolation of CS activity, and then held in place by the rubber platform. Recordings were made following completion of the electrode array. For further details on the electrode implantation procedure, see Sasaki et al. (1989).

All neuronal activity was recorded using a multichannel recording system (MultiChannel Systems, Germany) with a 25 kHz/channel sampling rate, gain of 1000x, and band pass filters set at 0.1 or 0.2–8.0 kHz (somatic level recordings, low cutoff of 0.1 kHz; dendritic level recordings 0.2 kHz). For the multielectrode recordings, CS activity could be discriminated using a single voltage level threshold, as simple spike activity was not detected because of the superficial placement of the electrodes. Recordings obtained at the PC somatic level contained both simple spikes and CSs, and therefore the entire record was spike-sorted offline to separate the two spike types.

### Zebrin experiments

Zebrin band location was used for grouping PCs, because PCs located in the same band receive input from the same region of the IO, and thus will likely have functionally related CS activity. The multielectrode recordings of CS activity used here were used in a previous study that described the relationship between the zebrin bands and the patterns of CS synchrony, and details about the localization of PCs to specific zebrin bands can be found there (Sugihara et al., 2007). In brief, spontaneous crus IIa CS activity was recorded from arrays of PCs. Following the recording session (20 min), alcian blue dye was injected into the cerebellar cortex at the corners of the array to mark their locations, the animal was perfused, and the cerebellum was then stained for zebrin. The locations of the PCs in the array were then plotted on zebrin maps of the cortex using the dye marks as fiduciary points.

### Cerebellar nuclear cell recordings

Convergence onto the same nuclear cell was used as the criterion for grouping PCs. In these experiments a multielectrode array was implanted on crus IIa and a single microelectrode was used to search for cerebellar nuclear neurons once the array was completed. Upon good isolation of a nuclear cell, the activity of all cells was typically recorded for 20 min. The subset of PCs in the recording array that synapse with the nuclear cell was then identified using cross-correlation analyses. The detailed recording methods and analyses related to establishing synaptic connectivity have been published previously (Blenkinsop and Lang, 2011). In brief, a significant negative deflection in the CS-triggered correlogram that occurred at a latency of 1–5 ms after the CS onset was taken as evidence for a synaptic connection between the PC and cerebellar nuclear cell being recorded.

### Climbing fiber reflex experiments

Cerebellar white matter stimulation elicits both a direct CS response in PCs, due to orthodromically conducted action potentials along the olivocerebellar axons from the stimulation site, and a longer latency reflex response, mediated by the antidromic spikes that travel back to the IO and cause electrotonic spread of current, via gap junctions, to other IO neurons, that in turn generate spikes that travel back to the cerebellum to evoke the reflex CSs (Eccles et al., 1966a; Llinás et al., 1974; Sotelo et al., 1974; Blenkinsop and Lang, 2006; Marshall et al., 2007). Here we will analyze the waveforms of the reflex responses that were recorded for a previous study in which the response distribution and its dependence on gap junction coupling of IO cells were reported (Blenkinsop and Lang, 2006). Details of the methods can be found in that report. However, essentially, the multielectrode array was implanted on crus IIa as described earlier, and a bipolar stimulus electrode was lowered through lobule crus I to a depth of 1–2 mm into the cerebellar white matter. Approximately 300 current pulses (100–200 μs, 50–500 μA) were given in each experiment to evoke CS responses.

### Intra-IO injection experiments

Picrotoxin (1–2 mg/ml), gabazine (1 mM), or carbenoxolone (500 μM) dissolved in 0.9% saline or Ringers solutions was injected into the IO to manipulate CS synchrony levels (Lang et al., 1996; Lang, 2002). In these experiments, a multielectrode array was used to record CS activity. After baseline CS activity was recorded for one or more 20-min control periods, an electrode was lowered from the dorsal surface of the medulla to the region of the IO, guided by stereotaxic coordinates (Paxinos and Watson, 1998). When IO multi- or single-unit activity was observed through the electrode, a short (5 min) recording period was obtained, and a correlogram of the IO activity with the CSs was generated for each PC in the array. A clear peak in at least some of the correlograms was used as the criterion for identifying the desired injection site within the IO (i.e., the part of the IO that projects to crus IIa being recorded). The electrode was then removed and an injection pipette was lowered to the same coordinates. An injection of approximately 1 μL of drug solution was then made over 5 min [for example, see Figure 1C of Blenkinsop and Lang (2006)]. CS activity was then recorded for 20-min periods.

### Data analysis

#### Variance analysis

Because spikelets are not always unambiguously distinguishable from baseline noise fluctuations, we developed a method for characterizing their parameters indirectly by using the variance associated with the CS waveform. Recordings where the 0.1 kHz lower limit of the bandpass filter was used (somatic recordings) were first high pass filtered using the FIR filter in Igor Pro (Wavemetrics) with a Hanning window with the transition band frequency limits set to 400 and 500 Hz. This was done for the recordings in the cerebellar nuclear recording, climbing fiber reflex, and urethane experiments. The recordings for the zebrin and pharmacological experiments were not refiltered. Variance, σ^2^, of the recording signal for a specified time window containing n sample points is defined in the usual manner:

(1) $$\sigma^{2}=\frac{1}{n-1}\sum_{i=1}^{n}{\left(y_{i}-\bar{y}\right)}^{2}$$

where *y*_*i*_ is the value of the recording at time point *i*, and *y* is the average value of the *y*_*i*_ for the window.

**Figure 1:**
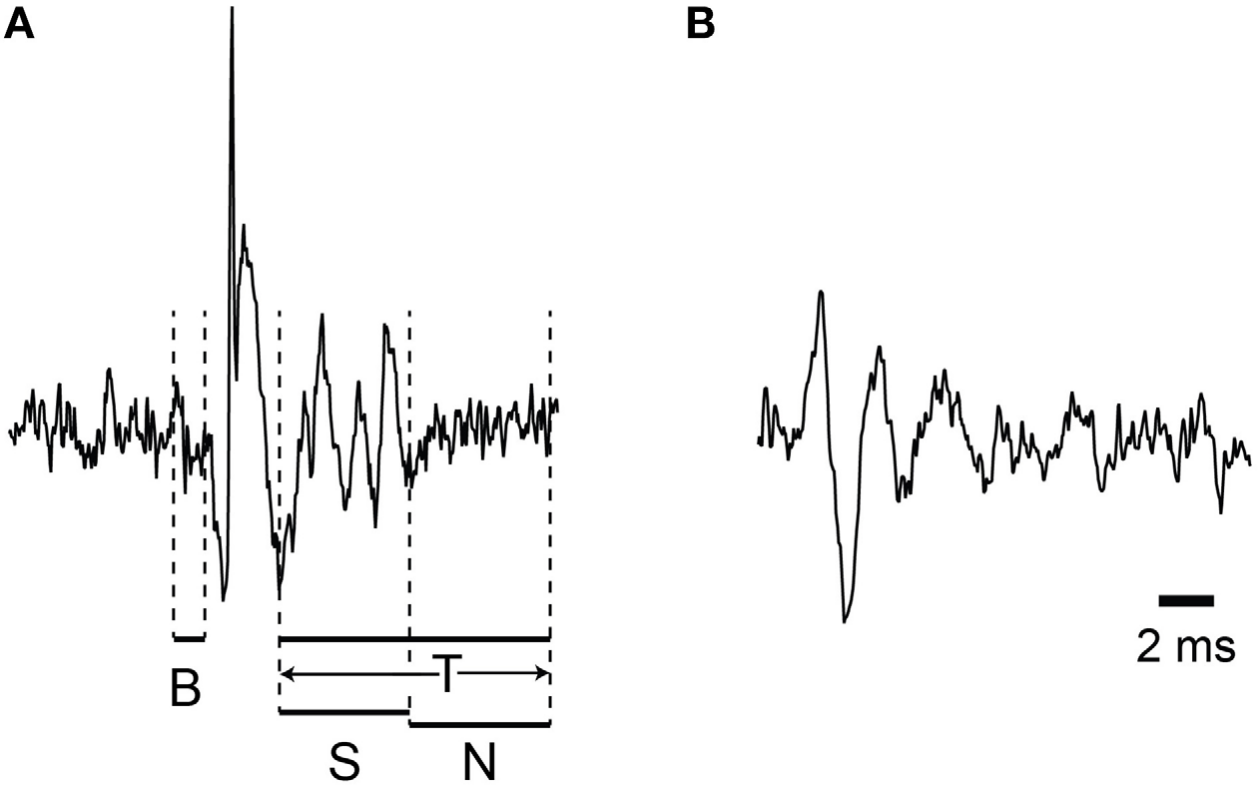
**Demonstration of the windows used for variance measurements**. The windows used to measure signal variance are illustrated: B (baseline), S (spikelet), N (non-spikelet), and T (total). **(A,B)** each present a sample CS waveform recorded from PC soma and dendrite.

We define several time windows for which variance measurements will be made (Figure 1A). The first is the total spikelet window (T), which is a fixed duration window that starts at the termination of the initial spike, and lasts long enough so that all (or nearly all) spikelets from any CS from the recorded PC will occur within it. The duration was chosen by first visually inspecting an overlay of all CSs and/or an average of all CSs from a PC to determine the approximate times of appropriate start and end points for that PC. The difference in these points set the duration of the T window. The exact duration used was not critical (similar results were obtained when the duration was varied by several milliseconds in test cases). However, if a PC had a few very long outlier CSs, they were excluded from the analysis in order to avoid making the T window excessively long, as that decreased its sensitivity for distinguishing between the vast majority of CSs.

For most CSs the T window can be divided into two successive portions, spikelet (S) and non-spikelet (N), where the dividing line is the time of the peak of the last spikelet plus half an interspikelet interval. This division point varies from CS to CS, and thus the S and N windows are variable, but their sum is fixed and equal to the time of the T window. Finally, we define baseline periods as those from times surrounding the CSs when no spike activity is present (i.e., either a time period before the CS starts or a period that starts later than end of the T window).

Depending on the analysis window there are several potential contributions to the overall variance in the recording. For the S window two signals contribute to the overall variance, the spikelet and the baseline noise signals. We assume that these are independent, and since the variances of two independent signals sum (Wonnacott and Wonnacott, 1977), σ^2^_*S*_ = σ^2^_*spikelet*_ + σ^2^_*base*_. By definition, σ^2^_*spikelet*_ is that due to only the spikelets themselves. The σ^2^_*base*_ includes all other signals, including the background noise of the electronics and any physiologically-generated electric activity not due to the spikelets. For the N period the variance is simply σ^2^_*base*_.

The signal during the T period is a combination of the signals during the S and N periods, and so, in general, its variance, σ^2^_*T*_, depends on σ^2^_*S*_ and σ^2^_*N*_ as well as the mean values of the S and N signals according to Equation (2) (Frühwirth-Schnatter, 2006):

(2) $$\sigma_{T}^{2}=\sum_{i}f_{i}(\sigma_{i}^{2}+{\left(\mu_{i}-\mu_{T}\right)}^{2})$$

where *i* = S and N, μ_*T*_ is the mean of the signal over *T*, μ_*i*_ is the mean of the signal over period *i*, and *f*_*i*_ is the fraction of T that corresponds to period *i*. However, note that if the baseline noise and spikelet signals have the same mean amplitude, the overall variance is simply the weighted sum of the variances of the individual S and N periods.

Finally, we define a modified total variance, σ^2^_T^*^_, where the contribution due to the difference in the means of the S and N periods is subtracted, by Equation (3).

(3) $$\sigma_{T^{*}}^{2}=\sigma_{T}^{2}-\sum_{i}f_{i}{\left(\mu_{i}-\mu_{T}\right)}^{2}=\sum_{i}f_{i}\sigma_{i}^{2}$$

#### Testing goodness of linear fits to variance plots

In many of the analyses the variance of each of the time windows defined above was plotted against particular variables, such as spikelet number or synchrony level. Least squares regression lines were then fit to these scatterplots to assess the relationship between the variables. A linear model was chosen, in part, because of the linear summation properties of the variances. The goodness of the regression model was tested by comparing the mean sum of squares related to within group or pure error (PE) and the error due to deviation from linearity or lack of fit (LOF), as defined according to Equations (4) and (5) (Brook and Arnold, 1985; Zar, 1999). The sum of the squares (SS) of the PE is:

(4) $$SS_{PE}=\sum_{i=1}^{k}\sum_{j=1}^{n_{i}}{\left(y_{ij}-{\bar{y}}_{i}\right)}^{2}$$

where there are *n*_*i*_ observations at the ith value of the independent variable, *k* different values of that variable, and where *y*_*i*_ is the average of the *y*_*ij*_ at the ith value of that variable. The SS_*LOF*_ is then obtained by subtracting the PE from the total error:

(5) $$SS_{LOF}=\sum_{i=1}^{N}{\left(y_{i}-\overset{⌢}{y_{i}}\text{​}\right)}^{2}-SS_{PE}$$

where the total error is the sum of the squares of the residuals about the regression line, $\overset{⌢}{y_{i}}$ is the predicted value of *y*_*i*_ from the regression equation, and *N* is total number of observations. The mean SS (MSS) for PE and LOF are then obtained by dividing them by their respective degrees of freedom: *MSS*_*PE*_ = *SS_PE_*/(*k* − 2) and *MSS*_*LOF*_ = *SS_LOF_*/(*N* − *k*), and the ratio MSS_LOF_/MSS_PE_, provides an F-statistic that can then be used to test the goodness of fit of the model (Brook and Arnold, 1985; Zar, 1999).

#### Counting spikelets

Spikelets were counted mainly in recordings with high signal-to-noise ratios in which separation of spikelets from noise fluctuations in individual traces was possible. To count spikelet numbers, all CS waveforms were first automatically processed by a custom-written procedure in Igor Pro (Wavemetrics, Portland, OR), which detected all deflections with a peak-to-trough level exceeding a pre-defined threshold. The counts were then manually verified, and only a small portion was adjusted with necessary deletions and/or additions (median percentage: 8%).

#### Synchrony analysis

In all analyses of synchrony, the time of the CS was defined as its onset. For a number of the analyses the level of synchronous CS activity was analyzed spike by spike. This was done by taking the time of the reference CS and determining whether CSs occurred in the other PCs in its group within a specified time window surrounding that time. The time windows for defining synchrony were either 1 or 5 ms depending on the experiment. The 1 ms window was used except when too few highly synchronous events (i.e., synchronous CSs among a large percentage of the group members) occurred for the analyses to be performed. In several instances we ran the analyses using both definitions, and similar results were obtained.

For some analyses, it was necessary to define the average level of synchrony that a PC had with other cells over an entire recording session. In these cases, we quantified the level of synchronous activity using a cross-correlation coefficient, *C*(0), as described previously (Gerstein and Kiang, 1960; Sasaki et al., 1989). The spike train of a cell was represented by *X(i)*, where i represents the time step (*i* = 1, 2, …, *N*). *X(i)* = 1 if a CS onset occurs in the ith time bin, otherwise *X(i)* = 0. *Y(i)* was the same as *X(i)*, but for the reference cell. *C*(0) was then calculated as:

$$C(0)=[\sum_{i=1}^{N}V(i)*W(i)]/\sqrt{\sum_{i=1}^{N}V{\left(i\right)}^{2}*\sum_{i=1}^{N}W{\left(i\right)}^{2}}$$

where *V(i)* and *W(i)* are

$$V(i)=X(i)-\sum_{j=1}^{N}\frac{X(j)}{N},W(i)=Y(i)-\sum_{j=1}^{N}\frac{Y(j)}{N}$$

Unless otherwise stated, population statistics are given as mean ± *SD*. The regression lines in this and all subsequent figures were fit using least squares.

## Results

For investigating the relationship between CS waveform (in particular, the number of spikelets) and synchrony with extracellular recordings, the relatively small and variable amplitude of the spikelets presents an obstacle. CSs recorded from near the PC layer are shown in Figure 1A. Here the initial spike amplitude is approximately 10 times the baseline, but the larger spikelets are only about 2 times the baseline noise, and what may be smaller spikelets fall within the range of the baseline noise fluctuations. As a result, not all spikelets can be unambiguously identified in most recordings, even with recordings that have high signal-to-noise ratios. The issue is more problematic for multielectrode recordings from the molecular layer, where the single to noise ratio of most spikes is usually less than what it is when a single electrode, which can be continually repositioned, is used for recording (Figure 1B). As a result, although isolation of CSs is not an issue, distinguishing spikelets from noise fluctuations is often difficult and time consuming, and generally cannot be done with absolute certainty from the multielectrode recordings needed to measure population activity.

Thus, we used the variance of the recording signal during various time windows during the CS as an indirect way to detect changes in the CS waveform. Below we will first present results showing that the variance in the T window (Figure 1A), which corresponds to the time when spikelets may be present, is modulated by the level of CS synchrony. The rationale for using the T window is that the variance in it will increase linearly with the number of spikelets contained within it (assuming that spikelet shape doesn't also vary with number, i.e., that σ^2^_*S*_ is constant). This follows from Equation (2), that σ^2^_*S*_ > σ^2^_*N*_, and that an increase in the number of spikelets is reflected as an increase in *f_S_*. However, other factors may influence the variance during the T window, such as fields generated by synchronously active nearby neurons, and other assumptions may not hold. For example, the shape of the spikelets could, in fact, vary systematically with the total number of spikelets. These possibilities will also be addressed below. Lastly, direct evidence for changes in spikelet number with synchrony will be presented.

### Spikelet-related variance is correlated with CS synchrony levels within a zebrin compartment

To test the relationship between CS synchrony and CS waveform, we analyzed multielectrode recordings of CS activity from crus IIa PCs whose locations had been mapped onto zebrin II stained brains in a previous study (Sugihara et al., 2007). PCs located in the same zebrin band receive their climbing fibers from the same small region of the IO (Voogd et al., 2003; Sugihara and Shinoda, 2004; Voogd and Ruigrok, 2004). Thus, CS synchrony among PCs in the same zebrin band should reflect the coupling state of the local region of the IO that projects to that band.

**Figure 2:**
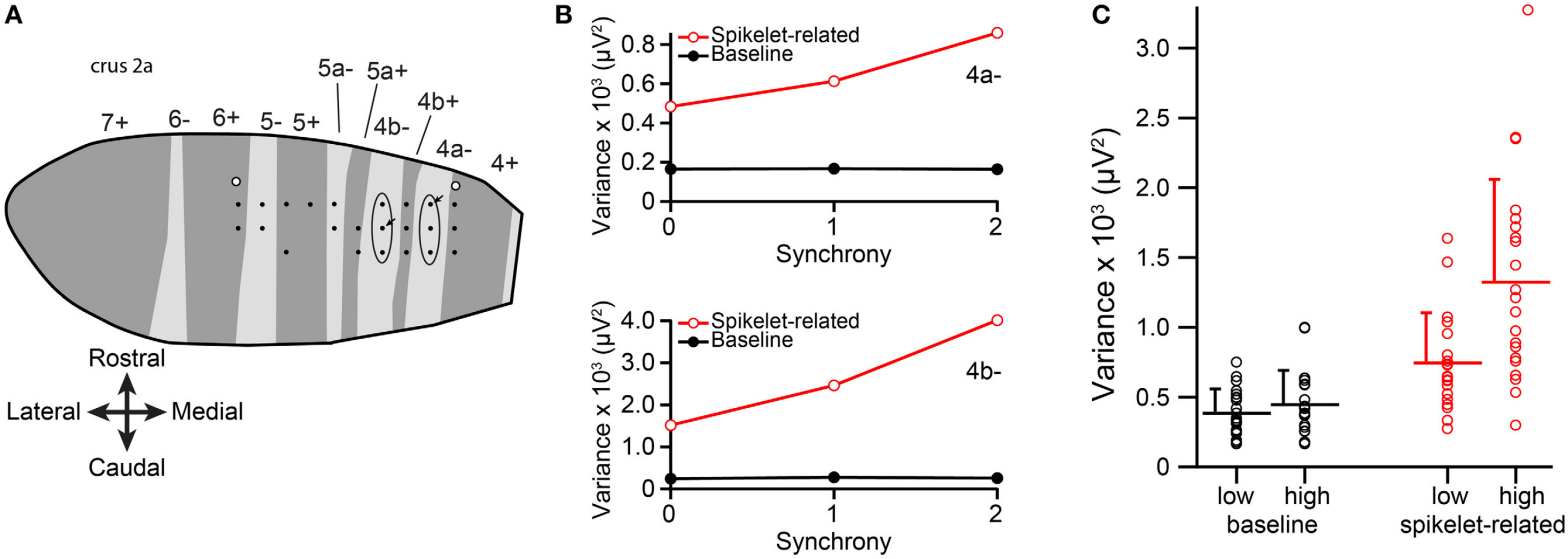
**Spikelet-related variance increases with CS synchrony among PCs within a single zebrin band. (A)** Plotting of electrode array from one experiment onto the zebrin map of crus IIa. Each black dot represents the position of an electrode in the array. Ellipses indicate the two PC groups whose CS activity was analyzed from this experiment. White-filled circles indicate locations of dye injections that were used to align the recording array with the zebrin map. **(B)** Plots of average variance as a function of group synchrony for the two PCs indicated by arrows in **(A)**. **(C)** Average variance for the lowest and highest synchrony levels for the population. Each circles represents data from one PC, bars indicate population averages, and error bars are one *SD*.

We analyzed data from three such multielectrode experiments. In total, seven groups of three PCs, where all of the PCs were located within the same zebrin band, were analyzed (*n* = 21 PCs). According to the nomenclature of Sugihara and Shinoda (2004), the PC groups were located in bands 4−, 4b−, 5−, 5+, 6−, and 6+. The plotting of PCs onto a zebrin map for one experiment is shown in Figure 2A. The two groups of PCs from this experiment that were analyzed are enclosed by ellipses.

To analyze the relationship between CS waveform and synchrony among the PCs in a zebrin band group, each CS was classified according to the level of synchrony in its group at its time of occurrence; i.e., according to how many other PCs (zero, one, or two) in the group also fired a CS within 5 ms of its onset. Spikelet-related variance (T window) and baseline (from a period just following the T window) variances were measured for each CS.

Averages of baseline and spikelet-related (T window) variances were computed for each cell at every available synchrony level. Plots of average spikelet-related variance as a function of synchrony level showed a positive relationship for almost all PCs, as shown for two PCs from two groups in one experiment (Figure 2B). Because only two or three levels of synchrony existed for any given PC, we tested whether there was a difference in average spikelet-related variance levels between the lowest and highest synchrony groups rather than doing regression analyses. In 81% of PCs (17/21) the spikelet-related variance of the highest synchrony level was significantly different, all greater, than that of the lowest synchrony level (*p* < 0.05). Of the other four PCs, three showed no significant difference and one had significantly larger variance in the lower synchrony group. In contrast, for baseline variance, either no statistical difference in baseline variance between the highest and lowest synchrony groups (20/21, *p* > 0.05, two-sided *t*-test) or a small negative relationship (1/21, *p* = 0.004) was found.

On a population level, the distributions of average spikelet-related variance for the highest and lowest synchrony levels were also different, whereas the distributions of baseline variances were not (spikelet-related, *p* = 0.0027; baseline, *p* = 0.33; Figure 2C). In sum, synchronous CSs were associated with higher spikelet-related variance.

### Spikelet-related variance correlates with synchrony among PCS that project to the same cerebellar nuclear cell

To investigate the correlation between spikelet-related variance and CS synchrony with more resolution in terms of synchrony levels, we used experiments from a data set in which CSs were recorded from PCs that all projected to the same cerebellar nuclear neuron (Blenkinsop and Lang, 2011). This criterion was used because cerebellar nuclear cells tend to receive input from PCs that are located within the same zebrin compartment (Chung et al., 2009; Sugihara, 2011), and thus can be used as a surrogate for zebrin in identifying PCs that receive climbing fiber input from the same region of the IO.

**Figure 3:**
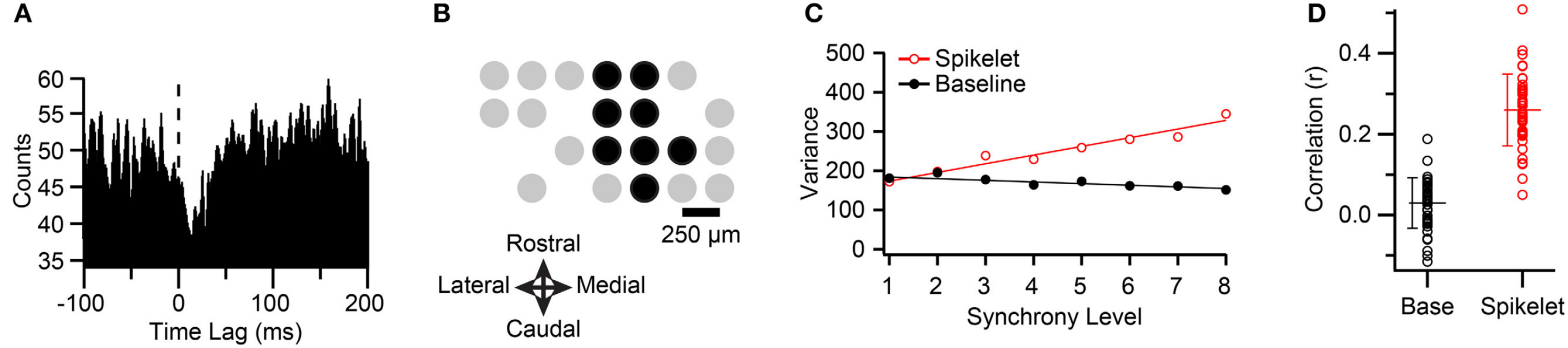
**Synchronous CSs among PCs projecting to the same cerebellar nuclear cell have higher spikelet-related variance. (A)** CS triggered histogram of cerebellar nuclear cell activity. Note the sharp reduction in activity just after the occurrence of the CS (time lag = 0 ms). Histogram was constructed using 1-ms bins, and smoothing by averaging each bin with its neighbors. **(B)** Schematic showing recording array arrangement. Each circle represents the relative position of an electrode on crus IIa. Black circles indicate PCs that projected to the cerebellar nuclear cell being recorded. **(C)** Plot of average spikelet-related (red) and baseline (black) variances as a function of synchrony level among the PCs indicated by black circles in **(B)** for one cell in the group (cell corresponding to the fifth circle of top row in **(B)**. A synchrony level (x-axis) of zero means that the CS occurred in the absence of CSs in any other PC in the group. Regression lines are fits to the entire data set for that cell. **(D)** Distribution of correlation (*r*) values between synchrony and variances for all PCs (circles). Population means indicated by horizontal lines, and error bars indicate the *SD*.

CS-triggered correlograms of nuclear cell activity were used to identify PCs that were synaptically connected to the nuclear cell, with the criterion being a sharp-onset inhibition of nuclear cell activity starting several milliseconds after the onset of the CS (Figure 3A, time lag = 0 ms, dashed line). This precisely timed inhibition, combined with the anatomy of the circuits, provides strong evidence of a direct synaptic connection between the PC and nuclear cell that were being recorded. For further details, including statistical tests of the significance of the inhibitory effect, see Blenkinsop and Lang (2011).

The CSs from six groups of crus IIa PCs so identified were analyzed, with the groups ranging in size from 4 to 9 PCs (mean 7.2 ± 1.7 PCs; *n* = 42 PCs total; *n* = 5 animals; note four PCs were part of two groups because two nuclear cells that were recorded in the same animal had partially overlapping PC groups). Figure 3B illustrates the arrangement of the PCs for one typical group, where the black circles indicate PCs that projected to the same cerebellar nuclear neuron and gray circles show the positions of the remaining PCs in the array.

For each PC, all of its CS were classified according to the level of synchrony in the group at the time of their occurrence using a 1-ms time window. The spikelet-related (T window) variance was also measured for each CS (defined in Methods). CSs were distributed over a range of 4–9 synchrony levels, depending on the PC. Although there was considerable scatter, a clear relationship between spikelet-related variance and synchrony could almost always be observed by plotting the average variance level as a function of synchrony within the group (Figure 3C, red circles).

To test the significance of the relationship between spikelet-related variance and synchrony, a scatterplot of these parameters was constructed from the CSs of each PC. Such plots showed a significant and positive correlation with synchrony for spikelet-related variance for 41/42 PCs (*p* < 0.02). The overall distribution of *r*-values is shown in Figure 3D (*r* = 0.26 ± 0.089, *n* = 42). Tests for linearity showed a good fit for most cells (MSS_LOF_/MSS_PE_; *p* > 0.05, *n* = 25/41 PCs). For the other PCs no dominant pattern to the deviation from linearity was found, with some cells showing supralinear increases at the highest synchrony levels, and some having curves that plateaued.

Next, to look for evidence of field effects that might contribute to the variance signal, we measured the baseline variance in the millisecond just prior to the onset of the CS. If fields due to synchronized activity from nearby cells were contributing to the signal, they should be present in the milliseconds preceding the CSs in the recorded cell, because “synchronized” CSs occur over a time window that spans at least several milliseconds (see Discussion for details).

Overall, the *r* values for the baseline (0.030 ± 0.062, *n* = 42) were much smaller than those for the T window. However, although the *r* value for the baseline was either not different from zero or negative for the majority of PCs (Figures 3C,D, black circles; *p* > 0.05, *n* = 20; *r* < 0 and *p* < 0.05, *n* = 3), for many PCs, a small but significant correlation was found (*r* > 0 and *p* < 0.05, *n* = 19). Thus, in at least some cells, fields may be contributing to the observed correlation between synchrony and the signal variance. However, for the 41 PCs with significant positive correlations for spikelet-related (T window) variance, the baseline *r* value was smaller than that for the T window. Moreover, we compared the slopes of the regression lines, and the line for the baseline variance was less steep than that for the T window for 37 of 41 PCs, with the difference being significant in 26 cases. Overall, excluding one outlier, the slope for the T window variance was on average 4–5 times greater than that for the baseline (T window: mean, 19.76 ± 35.62, median, 13.59; baseline: 4.43 ± 7.42, median, 2.18 μV^2^/synchrony level).

### Spikelet-related variance is correlated with the number of PCS responding to the climbing fiber reflex

We next investigated whether we could see the same relationship between variance and synchrony for CSs that were evoked using the climbing fiber reflex. The timing of such evoked CSs should occur randomly with respect to the ongoing state of the cerebellar cortex, and therefore should be evoked independent of direct effects of the state of the cerebellar cortex on the PC. Thus, the evoked CS waveforms should reflect factors related to the IO state, and not cortical activity directly. An indirect effect is still possible, as the cortical state can influence the state of the IO (Marshall and Lang, 2009; Chaumont et al., 2013), but the effect of changes in the IO state are what we are trying to demonstrate.

In each experiment, an array of electrodes was implanted into crus IIa to record CS activity (*n* = 51 PCs, 3 animals evoked via the climbing fiber reflex). The reflex was triggered by electrical stimuli (*n* = 300) delivered to the cerebellar white matter via a bipolar electrode implanted into crus I. Nine PCs were chosen for analysis because their reflex responses allowed individual spikelets to be counted (see later Section “Spikelet Count Correlates with CS Synchrony”). The average response rate among these nine cells was 24.6 ± 7.9% (range, 15–38%).

**Figure 4:**
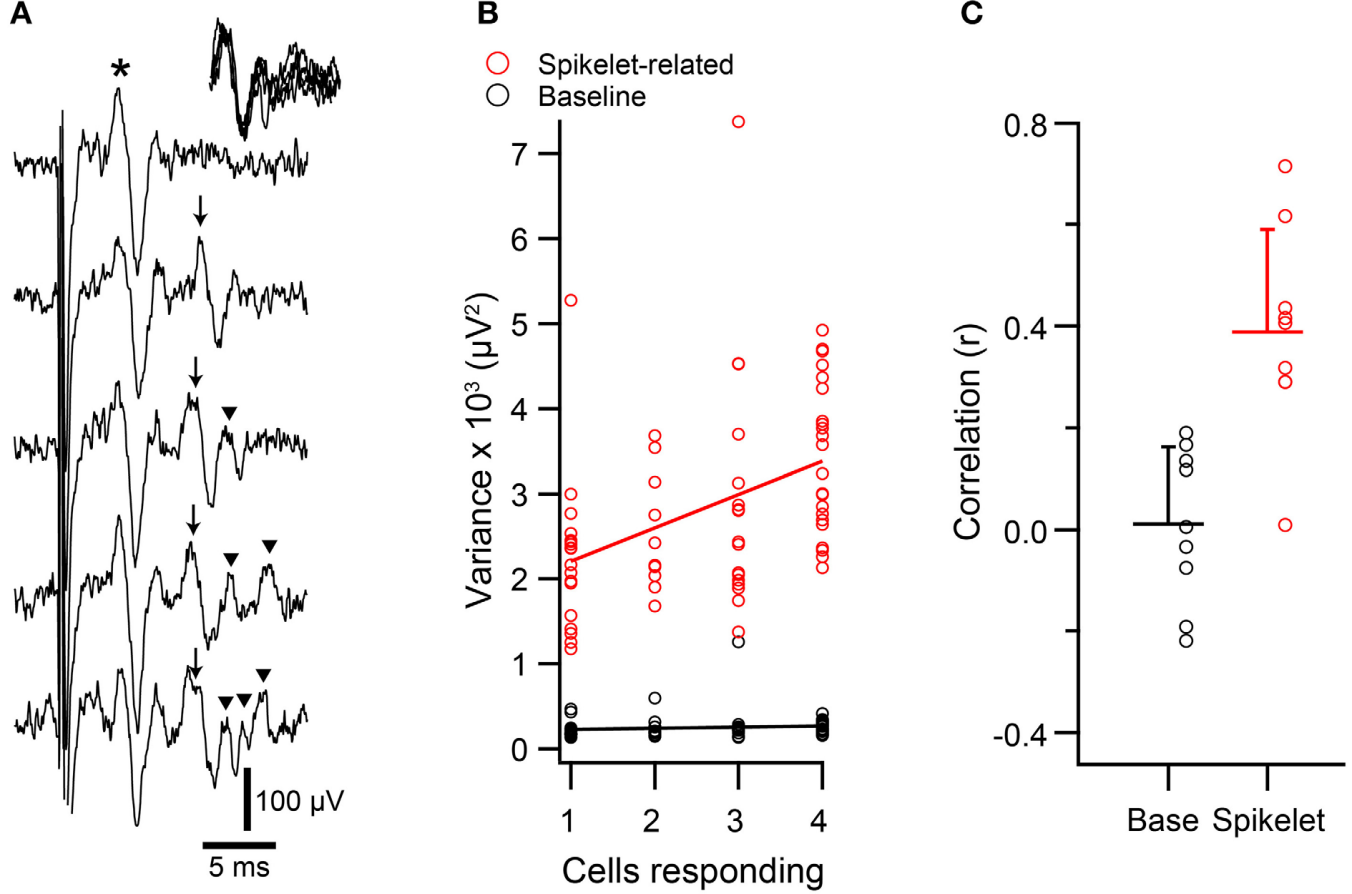
**Spikelet-related variance in climbing fiber reflex evoked CSs is correlated with size of responding population. (A)** Extracellular recordings of evoked responses, which showed both an initial direct response (*) and a reflex response (arrows). In the top trace only a direct response is present, whereas the remaining traces have both responses. Note the variable number of spikelets in the reflex responses (initial spike and each spikelet indicated by an arrow). Inset at upper right: overlapping of reflex response from bottom four traces, aligned to the initial spike in the reflex response. **(B)** Scatterplot of baseline (black) and spikelet-related (red) variance vs. the number of PCs that responded to the reflex for a representative PC. In this experiment the data were analyzed from four PCs, all of which showed significant reflex response percentages (17, 22, 23, and 29%). Circles represent the data from trials in which a response was evoked for this PC (one cell responding means that a response was evoked in only this PC). **(C)** Average correlation values for the relationship between variance and number of responding cells for baseline (black) and spikelet-related (red) variance. Each circle represents the data from one cell. Horizontal bars indicate mean of population. Error bars show 1*SD*.

Figure 4A shows the responses of a crus IIA PC to five electrical stimuli applied to the white matter core of crus I. In each case the stimulus evoked a short-latency direct CS response (Figure 4A, indicated by “*”) that is due to purely axonal conduction along the branches of the olivary axons. In most cases (bottom four traces), the direct response was followed by a climbing fiber reflex response (indicated by arrow and arrowheads) whose reflex arc involves spread of current among IO neurons via gap junctions, and thus, whose spatial distribution reflects the state of electrical coupling among IO neurons (Llinás et al., 1974; Sotelo et al., 1974; Blenkinsop and Lang, 2006; Marshall et al., 2007).

The direct response was relatively constant, and in this cell, usually consisted of a single negative deflection with one or two surrounding positive peaks. In contrast, the waveform of the reflex CS contained varying numbers of spikelets whose shape and timing could also vary. However, note that the initial spike of the reflex response was relatively constant, as can be seen by aligning the traces to its onset (Figure 4A, traces in upper right corner).

To investigate whether variation in the spikelet portion of the reflex response waveform was related to the electrical coupling state of the IO, T window variance was measured. Scatter plots were then constructed to compare the variance of each reflex response of a PC to the number of PCs in the array showing a response to the corresponding stimulus (Figure 4B, red circles). Almost all PCs (*n* = 8/9) showed a significant positive correlation between spikelet-related variance of a reflex CS and the number of PCs responding to the same stimulus (*p* < 0.05). In contrast, for the baseline variance, which was measured during the time just preceding the electrical stimuli, no significant correlation was found (*n* = 0/9; Figure 4B, black circles). Overall, the average correlation of spikelet-related variance with the number of cells responding was significantly different from zero, whereas that of the baseline value was not (Figure 4C; spikelet-related, *r* = 0.39 ± 0.20, *n* = 9, *p* = 0.0004; baseline, *r* = 0.01 ± 0.15, *n* = 9, *p* = 0.84). Moreover, the average spikelet-related correlation was significantly higher than that of the baseline (*p* = 0.0018, paired *t*-test).

### Spikelet-related variance and CS synchrony are correlated under urethane anesthesia

**Figure 5:**
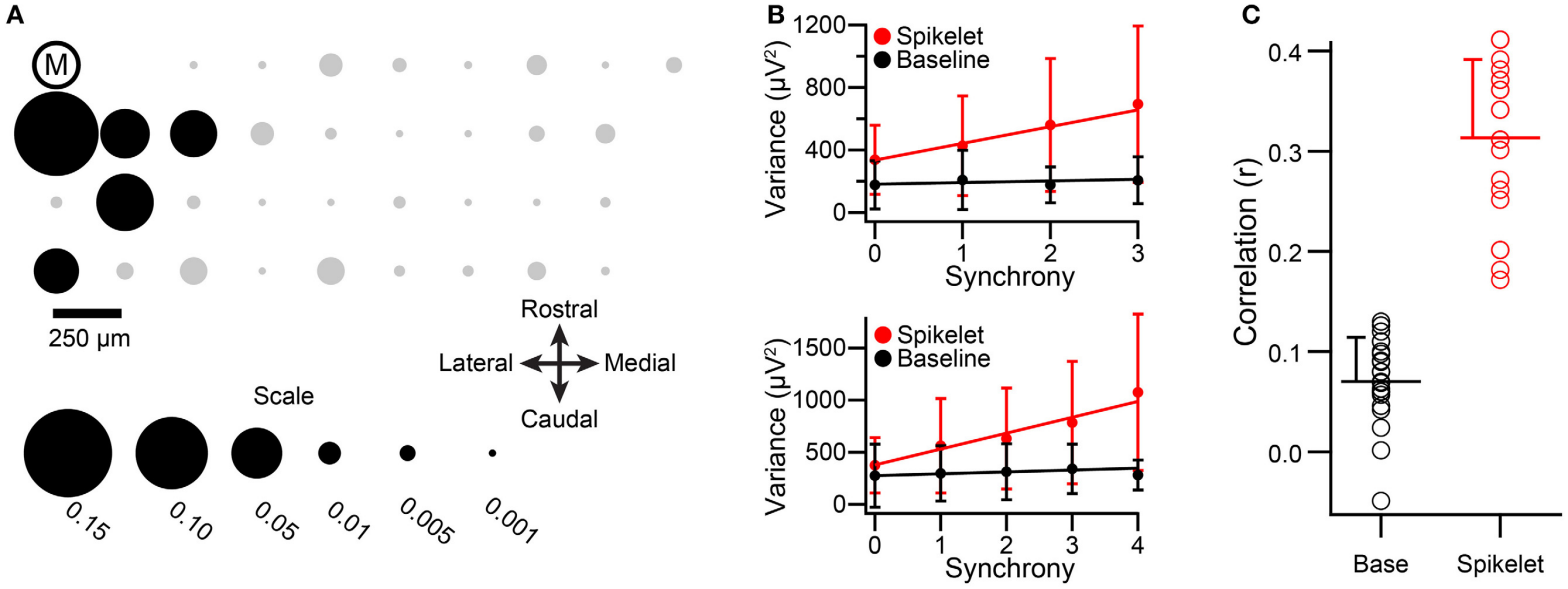
**Relationship between spikelet-related variance and CS synchrony holds under urethane anesthesia. (A)** Bubble plot representing the synchrony distribution with respect to cell M. The relative positions of the recorded PCs in the array are indicated by circles, and the encircled “M.” The areas of the circles are proportional to the synchrony between CSs of cell M and the other PCs in the array. Note that cell M's activity is strongly correlated mainly with neighboring cells. Black circles along with cell M comprise the cell group that was analyzed. **(B)** Plots of average variances during the CSs and the baseline periods as a function of CS synchrony among group members. Top graph is for cell M. Bottom graph is for another group member (indicated by black circle in third row, second column of the array). Regression lines are fits to the entire set of CSs for each cell, not the average points that are plotted. Error bars are one *SD*. Synchrony defined using a 5 ms bin. **(C)** Distribution of correlation values for the relationship between synchrony and variance for spikelet-related and baseline periods. Circles indicate individual cell *r* values, horizontal lines show distribution means, and error bars indicate the *SD*.

The above results demonstrating a correlation between spikelet-related variance and CS synchrony were all obtained in ketamine/xylazine anesthetized animals. To rule out the possibility that this relationship is specific to the ketamine/xylazine state, we analyzed data from urethane anesthetized animals (*n* = 21 PCs, 2 animals). Multielectrode recordings of spontaneous CS activity were obtained for 20-min periods. In these experiments we did not have zebrin stained tissue or simultaneous recordings of cerebellar nuclear cells, and so cell groups were formed as a localized cluster of PCs that had synchronized CS activity. The spatial distribution of synchrony under urethane is similar to that found under ketamine; that is, synchronous activity is most common among PCs aligned in the same rostrocaudal strip of cortex (Blenkinsop and Lang, unpublished results), and this rostrocaudal pattern aligns with the zebrin banding and PC-nuclear cell projection patterns (Sugihara et al., 2007; Sugihara, 2011). Therefore, these groups should be quite similar to those formed by the other two criteria. An example of the synchrony distribution with respect to one cell (cell M) of the recording array is shown in the bubble plot of Figure 5A, where the area of each circle represents the synchrony between the CSs of cell M and those of the PC at the location of the circle. Because synchrony levels in the urethane experiments were somewhat lower than those in the ketamine ones, a 5-ms window was used to define synchrony in order to obtain enough synchronous events that included a large percentage of PCs in the group.

In total, the CS activity from four groups of 5–6 PCs (two groups from each animal) were analyzed for the relationship between spikelet-related (T window) variance and synchrony levels. As was observed in the experiments where ketamine/xylazine anesthesia was used, a clear correlation between spikelet-related variance and the level of synchrony among group members was found. This is illustrated in Figure 5B for two cells from a PC group (the group comprises cell M and those PCs indicated by black circles in Figure 5A). For both PCs, the average spikelet-related variance (red circles) rises with synchrony level, whereas baseline variance (black circles) remains essentially constant. In these experiments the baseline was measured for a 4-ms period starting 10 ms after the onset of the CS, shortly after the end of the T window, because the activity preceding the CS onset was not recorded.

Almost all PCs showed a significant positive correlation between spikelet-related variance and synchrony level (Figure 5C, red circles; *p* < 0.05, *n* = 21/22; note, the activity from one PC was analyzed as part of two groups, giving a total n of 22). In contrast, for baseline variance, 7/22 PCs showed no significant correlation (*p* > 0.05), and while the remaining 15 PCs did show a statistically significant correlation, the values were small in comparison with those of spikelet-related variance (Figure 5C, black circles; baseline all cells: *r* = 0.072 ± 0.042, *n* = 22, baseline cells with significant r: *r* = 0.076 ± 0.045, *n* = 15; spikelet-related, all cells, 0.31 ± 0.077, *n* = 22). Furthermore, in those cases where a significant positive correlation of baseline variance occurred, the correlation of synchrony with spikelet-related variance was always greater than that with baseline variance (*n* = 15/15). In sum, the results were obtained under urethane and ketamine/xylazine anesthesia were quite similar.

### Pharmacological manipulations of CS synchrony produce corresponding changes in spikelet-related variance

To test whether the correlation between synchrony and spikelet-related variance reflects a direct causal relationship, we first manipulated CS synchrony levels by injecting a GABA-A receptor antagonist (either picrotoxin or gabazine) into the IO. Such injections have been shown to increase CS synchrony *in vivo* (Lang et al., 1996; Lang, 2002) and to increase synchronization of IO activity in brainstem slices (Leznik et al., 2002), effects that are likely due to increased electrical coupling within the IO (Onizuka et al., 2013; Lefler et al., 2014).

**Figure 6:**
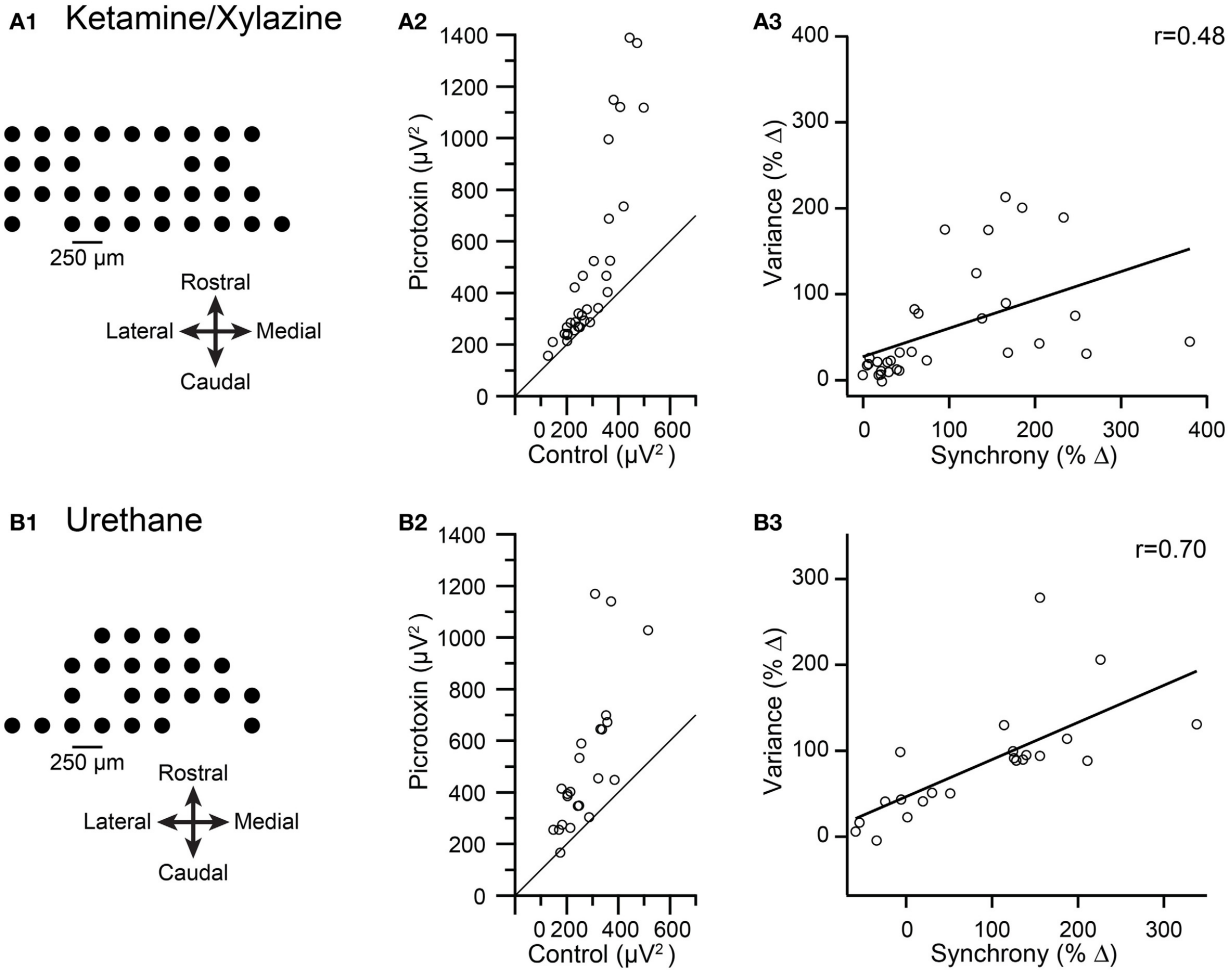
**Pharmacologically-induced increases in CS synchrony also increase spikelet-related variance**. Results from recordings made under ketamine/xylazine **(A)** and urethane **(B)** are shown. **(A1,B1)** Schematics showing recording arrays. **(A2,B2)** Plot of average spikelet-related variance in control (x-axis) vs. following an injection of picrotoxin into the IO (y-axis). Each circle represents the data from one PC in the array. Diagonal line in each plot is the line y = x. **(A3,B3)** Plots of percent change in variance between control and picrotoxin conditions vs. the percent change in synchrony between these conditions for each PC. Regression lines are plotted and *r* values are indicated.

Data were analyzed from three multielectrode experiments in which crus IIa CS activity was recorded. In two experiments ketamine/xylazine anesthesia was used (*n* = 50 PCs), and in the other urethane was used (*n* = 23 PCs). The spatial arrangements of the electrode arrays from two of these experiments are shown in Figures 6A1,B1. In all three experiments, injection of picrotoxin or gabazine into the IO increased CS synchrony (1 ms time bin) when measured across all PC pairs in the array (ketamine/xylazine: 70 and 40%; urethane: 88.6%). The average spikelet-related (T window) variance was calculated for the CS activity of each PC in control and after injection of a GABA-A antagonist, and then these variances were plotted against each other (Figures 6A2,B2). Across all three experiments, 68/73 cells showed an increase in variance. For the experiments in Figures 6A2,B2, the consistency of the effect is shown by almost all the data points being above the y = x line in the plots. The change in variance was significant in all three experiments (ketamine/xylazine, *p* = 0.0002, both experiments; urethane, *p* = 2.7 × 10^−5^, paired *t*-tests).

To test the relationship between spikelet-related variance and synchrony further, we calculated the percent change in both synchrony level and spikelet-related variance from the control to the drug condition for each PC. For all three experiments a significant correlation existed between the change in synchrony and the change in variance levels (ketamine/xylazine: *r* = 0.48 and 0.54, *p* = 0.013 and 0.02; urethane: *r* = 0.70, *p* = 0.0003). Scatterplots from two of the experiments are shown in Figures 6A3,B3 to illustrate the relationship.

**Figure 7:**
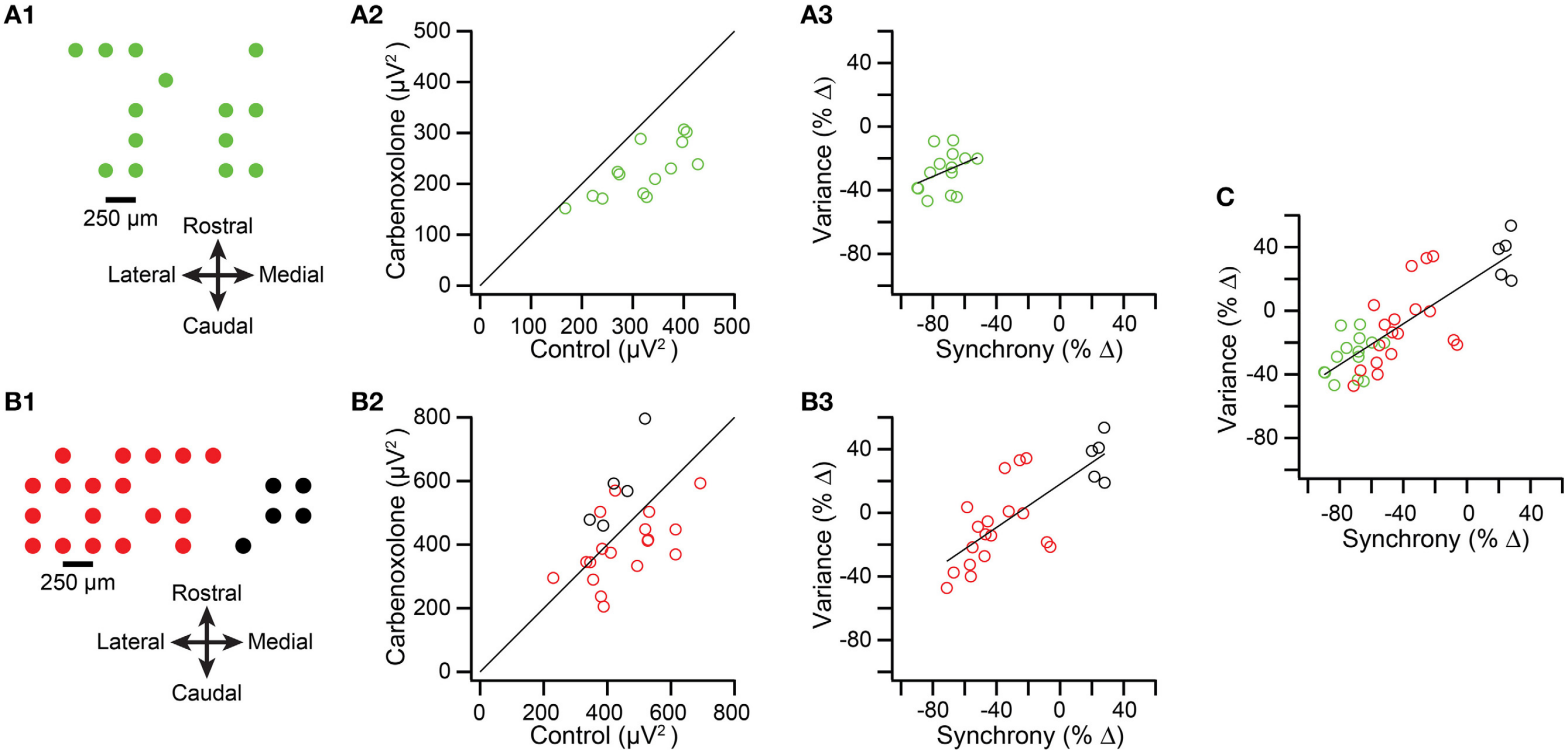
**Intra-IO injection of carbenoxolone reduces spikelet-related variance. (A1,B1)** Schematics showing layout of multielectrode recording arrays from two experiments. In the second experiment **(B1)** the red circles represent PCs whose CSs became less synchronous after carbenoxolone and the black circles represent PCs whose CSs became more synchronous. **(A2,B2)** scatter plot of spikelet-related variance in control vs. carbenoxolone condition. **(A3,B3)** Scatter plot of percent change in synchrony vs. percent change in variance between control and carbenoxolone conditions. **(C)** Data from plots **(A3,B3)** combined.

We next tested whether spikelet-related variance decreased when CS synchrony was reduced by injection of carbenoxolone, a gap junction blocker, into the IO in two experiments from a previous study (Blenkinsop and Lang, 2006). The recording arrays are shown in Figures 7A1,B1. Overall, the induced changes in synchrony and variance were highly correlated. In the first experiment the intra-IO injection produced a −72.5 ± 11.0% change in synchrony, with all PCs showing a reduction (*n* = 14 PCs; *p* = 6.4 × 10^−5^, paired *t*-test). Correspondingly, variance was reduced from control levels in every PC (−28.2 ± 12.7%; *p* = 1.5 × 10^−5^, paired *t*-test; Figure 7A2, all circles below y = x line). However, a scatterplot of the percent changes in synchrony and variance showed only a relatively weak correlation that was not significant (*r* = 0.37, *p* = 0.187; Figure 7A3).

The weakness of the correlation may be attributable to the strong and consistent reduction in synchrony caused by carbenoxolone in the experiment shown in Figure 7A, leading to both a floor effect and a relatively narrow range of synchrony changes over which to evaluate variance levels. This possibility is supported by the results of the second experiment (*n* = 23 PCs), in which carbenoxolone produced a more variable effect on CS synchrony, as was often the case (Blenkinsop and Lang, 2006). In this experiment, carbenoxolone reduced synchrony in most PCs (*n* = 18/23 PCs; Figure 7B1, red circles), but the magnitude of the reduction ranged widely, from just a few percent to almost 80% (Figure 7B3, red circles). Moreover, a minority of PCs had somewhat higher synchrony levels in the carbenoxolone condition (*n* = 5; Figures 7B1,B3, black circles). Note that these latter PCs were clustered on the medial edge of the recording array and likely received input from a different region of the IO than the remainder of the PCs, explaining the failure of the injection to reduce the synchrony level of their CS activity (Blenkinsop and Lang, 2006). Comparison of variance in the two conditions in the second experiment revealed that most PCs showing a reduction in synchrony also had a reduction in variance (Figure 7B2, red circles; note that most are below the y = x line), whereas all PCs showing an increase in synchrony also had an increase in variance (black circles). For the PCs that showed a reduction in synchrony, a modest but significant reduction in variance was found (−10.48 ± 34.1%; *p* = 0.029). However, in contrast to the first experiment, here the magnitude of the effect on variance varied widely, particularly when the PCs experiencing an increase in synchrony were included (Figure 7B3), and a strong correlation was found between the change in synchrony and variance in this case (*r* = 0.76; *p* = 2.8 × 10^−5^). Finally, when the PCs from both carbenoxolone experiments are combined, it can be seen that a clear correlation (*r* = 0.80; *p* = 2.8 × 10^−9^) between synchrony and variance is present for an extended range of synchrony changes (Figure 7C).

In sum, the results of the IO injection experiments show that manipulations that increase and decrease the electrical coupling of IO neurons lead to corresponding changes in spikelet-related variance.

### Measurement of field effects during the time of the spikelets

Comparison of the baseline and T window variance correlations with synchrony suggests that the T window variance correlation is largely due to changes in the CS waveform, as opposed to field effects from synchronized activity of neighboring cells. However, the baseline measurements were made just before or after the time of the spikelets, whereas, ideally, one would want to measure the baseline during the spikelet period itself. This is not possible when the PC being recorded is firing; however, it is possible to measure the contribution of these fields selectively by identifying synchronous CS events among neighboring cells during which the PC itself is not spiking (either CSs or SSs).

**Figure 8:**
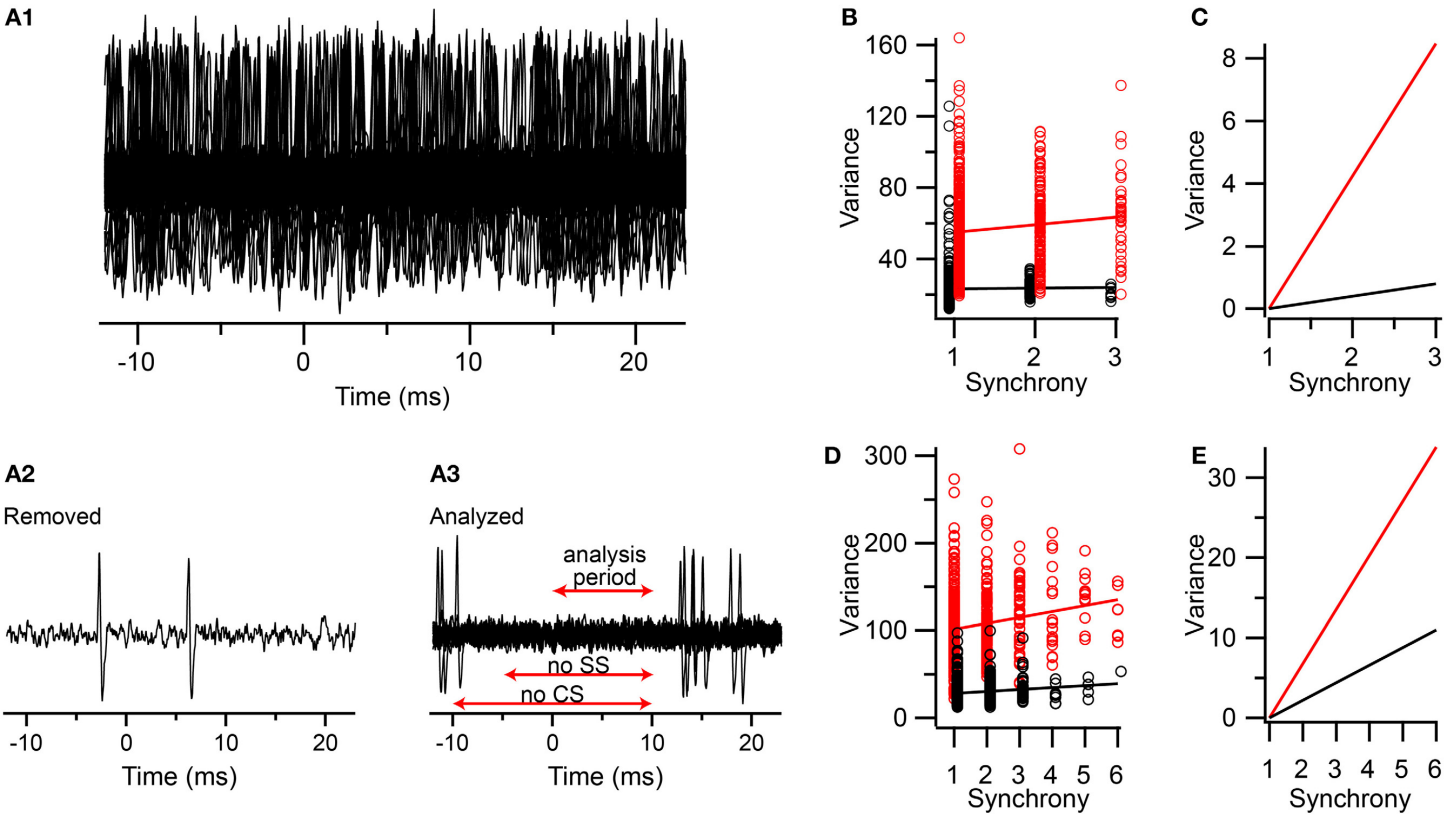
**Comparison of contributions of spikelets and fields from neighboring neurons to the correlation of synchrony and spikelet-related variance. (A1)** Segments of an extracellular recording from a PC all aligned to the times of the CSs in other PCs in a group of PCs whose CSs are synchronized. **(A2)** Example of one trace that was removed from set shown in **(A1)** because of simple spikes being present just before and during the analysis period **(A3)** The set of traces selected from those in **(A1)** in which no CSs or simple spikes were present during the time windows indicated (SS = simple spikes). **(B)** Plot of variance vs. CS synchrony among PCs in the group. Variance measurements during the 0–10 ms period in the traces with no spikes indicated by black circles. T window variance for the CSs for the PC whose traces were analyzed for fields indicated by red circles. **(C)** Replotting of regression lines shown in **(B)** after vertically aligning their left-most points. **(D,E)** Same as **(B,C)** respectively, but for a second experiment. Data markers in **(B**,**D)** are slightly offset along the x-axis for clarity.

We did this in two multielectrode experiments (one group from each experiment, *n* = 9 PCs/group) using urethane anesthesia. As was described above, the CSs for each cell in the group were classified according to the synchrony level at the time of their occurrence (5-ms window). Then one focal PC (the one with the highest signal-to-noise ratio) was selected for analysis. Next, segments of this PC's recording corresponding to the times of the CSs (from 10 ms prior to 10 ms after the onset) in each of the other cells in the group were made. Thus, for each synchrony level, a set of traces of the focal PC's activity were obtained, all aligned on the CSs of the other PCs (time *t* = 0 ms in Figure 8A). One such set is shown in Figure 8A1. In most traces the selected PC had spike activity (Figure 8A2), as shown by the denseness of the spikes when the traces are overlapped. To exclude spike-related activity from the selected PC, all traces with simple spikes between *t* = −5 and 10 ms or CSs between *t* = −10 and 10 ms were removed (the differing times reflects an attempt to preserve as many traces as possible for analysis while also avoiding contamination of the 0–10 ms period with spike-related activity). The remaining traces were then analyzed for their variance between *t* = 0 and 10 ms (Figure 8A3). The spikelet-related variance was also measured for the focal PC using a T window in order to compare it with that from the field only traces.

In both experiments the correlation between CS synchrony and the actual spikelet-related variance was significant, and in the range reported for the other experiments (Experiment 1: *r* = 0.11, *n* = 587 CSs, *p* = 0.007; Experiment 2: *r* = 0.19, *n* = 639 CSs, *p* = 1.7 × 10^−6^). The correlation for the field-only traces was smaller in each case, and only significantly different from zero in one experiment (Experiment 1: *r* = 0.017, *n* = 964 traces, *p* = 0.60; Experiment 2: *r* = 0.134, *n* = 809 traces, *p* = 0.00013). Thus, the fields can sometimes contribute to the correlation observed, consistent with the earlier baseline results. To test whether the correlation due to the fields could fully explain the one observed for the spikelets, we performed a regression analysis and compared the slopes of the two lines (Figures 8B–E). In both experiments the slope of the spikelet-related regression line is significantly steeper than that of the field-only regression line. This difference is most easily observed when the vertical displacement of the two lines is eliminated at their starts (Figures 8C,E), which shows that the spikelet-related lines were 10–11 and 3 times steeper than their field-related counterparts for the two experiments. Thus, only about 10 and 33%, respectively, of the effect of synchrony on the variance signal was explained by the contribution from the fields in these experiments.

The above results indicate that spikelet-related variance increases with synchrony. The potential causes underlying this relationship may relate to modulation of basic spikelet parameters. Thus, we now describe results related to how changes in these parameters affect spikelet-related variance.

### Spikelet-related variance is correlated with the number of spikelets in spontaneous CSs

**Figure 9:**
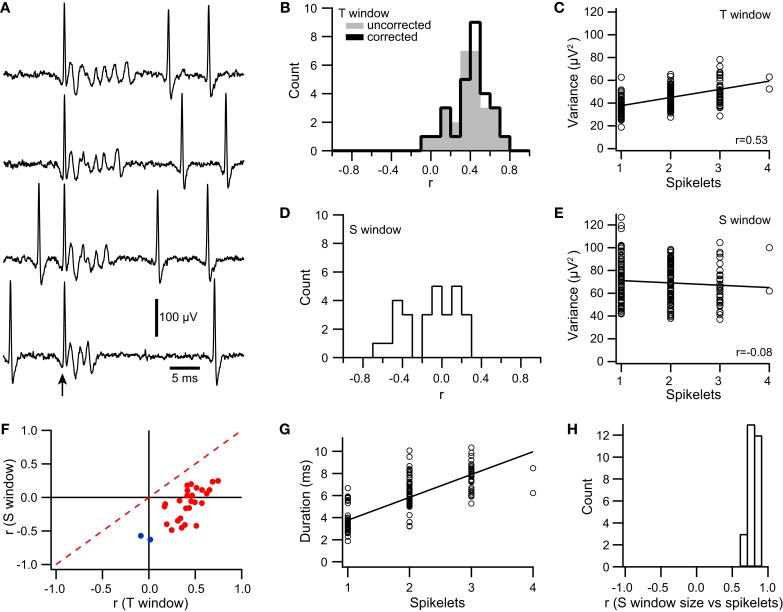
**Signal variance is correlated with the number of spikelets in a CS. (A)** Extracellular traces of a PC recorded near its soma. Traces are aligned to the onset of the CS (arrow). The number of spikelets varies between CSs. Simple spikes are also present before and after the CS depending on the trace. **(B)** Histograms showing distribution of correlation coefficient values (*r*) between the number of spikelets and spikelet-related variance (T window) using σ^2^_*T*_ (gray fill) and σ^2^_T^*^_ (black line). **(C)** Scatterplot of signal variance during the T window vs. spikelet count for a PC. Each circle represents the values for one CS. **(D,E)** Same as **(B**,**C)**, respectively except that the S window was used to measure variance. **(F)** Scatter plot of *r* values obtained by correlating spikelet number with variance for the S window and the T window. Significant *r* values for the T window are in red, those that are not are shown in blue. **(G)** Scatter plot of CS duration vs. number of spikelets for a typical cell, which generated CSs containing between 1 and 4 spikelets. **(H)** Histogram of *r* value between CS duration and number of spikelets for the somatically-recorded PC population.

To investigate how spikelet-related variance is influenced by spikelet number, we used a single electrode approach to make recordings of CS activity with high signal-to-noise ratios (*n* = 28 PCs total; 10 animals; crus IIa, *n* = 26; vermis lobule VIII, *n* = 2). These recordings were made at or near the level of the PC soma, as determined by the presence of simple spike activity and the predominantly positive polarity of the SSs and the initial spike of the CSs (Figure 9A). The high signal-to-noise ratio allowed semi-automated counting of spikelets with only minor manual corrections (see Methods).

For each PC, spikelet-related variance, σ^2^_*T*_, was measured using a fixed time window, the T window. For 93% of the PCs, a significant correlation was found between the variance and spikelet count (*r* = 0.42 ± 0.15; *p* < 0.05, *n* = 26/28; Figure 9B, uncorrected). A scatterplot was then constructed from the data of each PC, and a least squares regression line was fit to the data (Figure 9C). We tested for evidence of non-linearity by using the residuals to compare the pure error to the error due to the lack of fit of the linear model (Brook and Arnold, 1985; Zar, 1999). For 24 PCs having a significant correlation (two of the 26 such PCs were not analyzed because their spikelet number only varied between two values), no evidence for a non-linear dependency of variance on spikelet number was found in most cases (MSS_LOF_/MSS_PE_; *p* > 0.05, *n* = 19/24). The non-linear plots (*n* = 5/24; *p* < 0.05) were examined visually, and the deviation from linearity appeared to be due to a plateauing of the variance curve with increasing spikelet number.

### Changes in spikelet shape with number of spikelets in a CS

The linearity of the relationship found for most PCs between spikelet number and σ^2^_*T*_ suggests that shape of the individual spikelets (at least as measured by variance) doesn't generally co-vary with the number of spikelets. In contrast, the plateauing relationship observed in the remaining PCs suggests that in a minority of cases, spikelet size tends to vary inversely with number. That is, when more spikelets are present, their average size is smaller, resulting in progressively smaller increases in σ^2^_*T*_ with each additional spikelet.

To investigate this issue further, we assessed the dependence of spikelet shape on the number of spikelets in a CS by measuring variance using a variable time window whose duration was matched to that of the individual CS, the S window (Figure 1A). In contrast to σ^2^_*T*_, which is explicitly a function of both the shape and number of the spikelets, S window variance, σ^2^_*S*_, essentially depends on just the shape of the spikelets, because spikelets are present throughout the S window (i.e., the variance of the S window is approximately the average variance of the individual spikelets, and thus is independent of the number of spikelets). For the S window, the correlation of variance with spikelet number was positive in a few cases (*n* = 4, *r* > 0 and *p* < 0.05), but for the large majority of PCs, it was either not statistically different from zero (*n* = 13, *p* > 0.05) or negative (*n* = 11, *r* < 0 and *p* < 0.05) (Figures 9D,E). The median correlation coefficient was -0.35 for the 15 cells with significant correlation between the σ^2^_*S*_ and the spikelet number, and 0.03 for the 13 cells without significant correlation, whereas the overall median correlation coefficient was −0.09 (*n* = 28).

We then compared the correlation of spikelet number with σ^2^_*T*_ and σ^2^_*S*_. In all cases σ^2^_*T*_ showed a more positive (in one case less negative) correlation with spikelet number than did σ^2^_*S*_ (Figure 9F, all points are right of the dashed red y = x line). Furthermore, when r for σ^2^_*T*_ was large, r for σ^2^_*S*_ was close to zero, whereas as r for σ^2^_*T*_ moved toward zero, r for σ^2^_*S*_ became negative. Indeed, the two cells for which r for σ^2^_*T*_ was not significant had the two most negative *r* values for σ^2^_*S*_ (Figure 9F, blue points).

For cells showing a linear relationship between σ^2^_*T*_ and spikelet count, the correlation of σ^2^_*S*_ with spikelet count was close to zero (−0.03 ± 0.23; median = 0.03; *n* = 19). In contrast, for the cells having a plateau type curve with σ^2^_*T*_, the correlation with σ^2^_*S*_ tended to be negative (−0.22 ± 0.17; median = −0.11; *n* = 5). Although the correspondence is not absolute, it is consistent with the plateauing relationship between σ^2^_*T*_ and spikelet count observed for some PCs being due to a decrease in average spikelet amplitude as the number of spikelets increases.

The duration of the S window was then compared to the number of spikelets in order to assess whether spikelet duration varied with the number of spikelets. A strong linear relationship was found in all cases (*r* = 0.795 ± 0.065, *n* = 28, Figures 9G,H), indicating that average individual spikelet duration does not co-vary with spikelet number.

In sum, these results indicate that there is no consistent trend in average spikelet width and amplitude correlated with the number of spikelets for most PCs, but that for a significant minority of PCs, a tendency for spikelet amplitude to decrease with increasing spikelet numbers exists.

### Spikelet period has a distinct baseline variance

**Figure 10:**
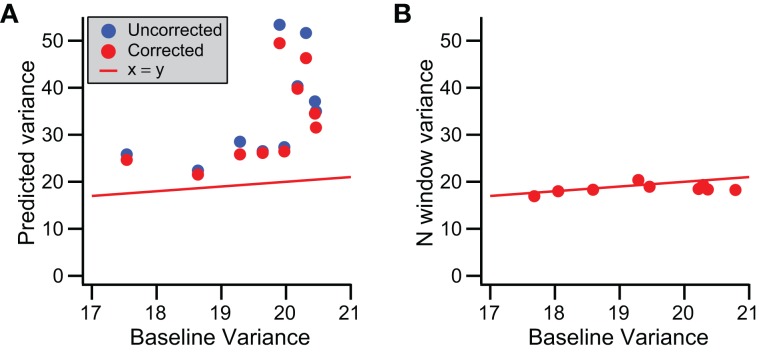
**Prediction of baseline variance during CS. (A)** Scatter plot of predicted baseline variance, obtained by extrapolating the regression line between number of spikelets and the spikelet-related variance to zero spikelet, vs. baseline variance measured from times surrounding the CS (outside the T window). The predictions were made from regression lines with either σ^2^_*T*_ (uncorrected, blue) or σ^2^_T^*^_ (corrected, red circles) vs. spikelet number. **(B)** Scatter plot of σ^2^_*N*_ vs. measured baseline variance. Red line indicates y = x in both **(A,B)**.

To test whether baseline variance is altered during the CS, we used the regression lines fit to the somatic recording data (Figure 9C). These regression lines describe spikelet-related variance, σ^2^_*T*_, as a function of spikelet number, and so extrapolating to zero spikelets gives a prediction of the baseline variance. We compared this predicted value with that measured directly from times just after the end of the T window in a subset of 15 PCs with a linear relationship between spikelet number and σ^2^_*T*_ (MSS_LOF_/MSS_PE_ ratio, *p* > 0.05) and the highest correlation coefficient values in the dataset (all *r* > 0.35). In all cases the predicted value was higher than the measured baseline (*p* < 0.001, paired *t*-test). This is shown in Figure 10A, where the predicted values (blue circles) all fall above the corresponding measured baseline values.

To understand the cause of this discrepancy, we first considered the possibility that the mean signal during the period when spikelets actually occurred (S window) was different from the mean for the later part of the T window, the N window, within which the spikelets were absent, as such a difference would increase the overall spikelet-related variance, σ^2^_*T*_, and thereby over-predict the baseline variance (see Equation 2). To test this possibility, we computed a corrected variance, σ^2^_T^*^_, for each CS using Equation (3), and carried out the above analyses with this new value. Correlation of σ^2^_T^*^_ with spikelet number produced similar, though slightly higher, *r* values (0.44 ± 0.15; Figure 9B, compare corrected and uncorrected histograms). Extrapolation of regression lines based on the relationship of σ^2^_T^*^_ to spikelet number to zero spikelets gave lower estimates of baseline variance (Figure 10A, red circles; *p* < 0.001, paired Wilcoxon signed rank test), but ones that were still well above the measured values from the surrounding times.

These results suggest that the baseline variance (estimated via extrapolation) is, indeed, different during the CS than at times when there is no CS activity. Thus, we next assessed whether this change occurs throughout the T window or whether it is limited to the S window. During the S window both baseline and spikelet signals are present, making it impossible to measure the baseline signal. Instead, we measured the variance for the N window, σ^2^_*N*_, to test whether it was different from the general baseline. For this analysis it was critical not to have any spikelet-related activity in the N window, so here the N window was defined as starting one interspikelet interval after the peak of the last counted spikelet (rather than half of an interspikelet interval). This delay prevented contamination from the trough of the last spikelet and its recovery. σ^2^_*N*_ was found to be statistically the same as the general baseline for almost all cells (14/15, *p* > 0.05; Wilcoxon rank sum tests). However, even though σ^2^_*N*_ was not statistically significant for individual cells, viewing the population data (Figure 10B) suggests that σ^2^_*N*_ has a slight tendency to fall below the baseline variance line, and indeed on a population level, this difference was significant (*p* = 0.0074, *n* = 15; paired *t*-test), indicating a small depression of variance immediately following each CS. Nevertheless, the match of σ^2^_*N*_ and the general baseline variances suggests that the change in the baseline variance during the T window occurs during the S window, that is, during the time when spikelets are actually occurring.

### Spikelet count correlates with CS synchrony

The above results provide evidence that CS waveform varies with CS synchrony, and are consistent with the change in waveform being due to changes in spikelet number with synchrony. However, they do not rule out the possibility that changes in spikelet size are responsible. Thus, to provide direct evidence for spikelet number varying with synchrony, we have examined both CF reflex evoked and spontaneous CSs in a few PCs recorded with a multielectrode array and where the spikelets were relatively large, making direct counting of spikelets possible.

**Figure 11:**
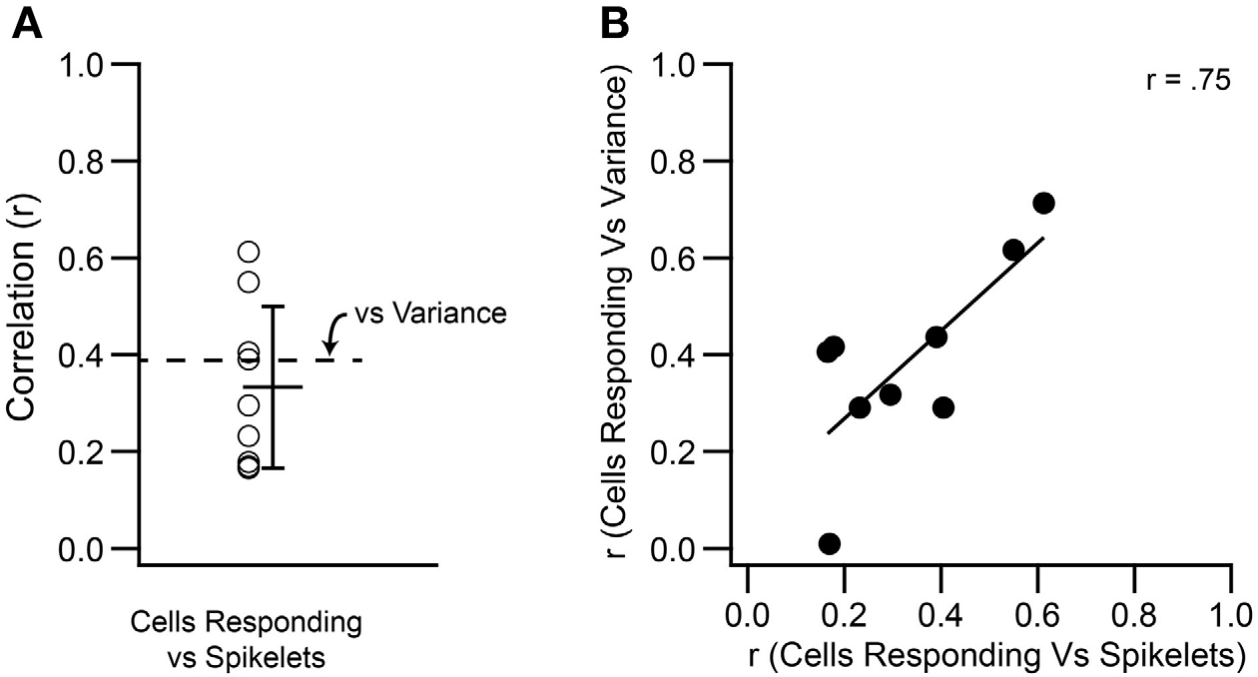
**Variance and spikelet number are correlated to the number of PCs showing climbing fiber reflex evoked CSs. (A)** Each circle shows, for a PC, the *r* value for the correlation between number of spikelets in an evoked CS and number of cells showing a reflex response to the same stimulus. Mean and *SD* are shown. The mean *r* value for the correlation between T window variance and number of responding cells is replotted from Figure 4 for comparison (dashed line). **(B)** Scatter plot showing the correspondence between the *r* values for correlation of variance and spikelets with the number of PCs responding to a stimulus.

Spikelets were counted for the climbing fiber reflex responses of 9 PCs. These were the same PCs as described in Figure 4, so that the correlation of both measures of the CS waveform (variance and spikelet count) to the size of the reflex response could be directly compared. Correlation of spikelet count with the number of PCs responding yielded significant correlations in all cases (*p* < 0.05). The correlation values obtained using spikelet counts (*r* = 0.33 ± 0.17) were not statistically different from those obtained with variance measurements (*p* = 0.25, paired *t*-test; Figure 11A), and were well matched to each other across the population (Figure 11B).

**Figure 12:**
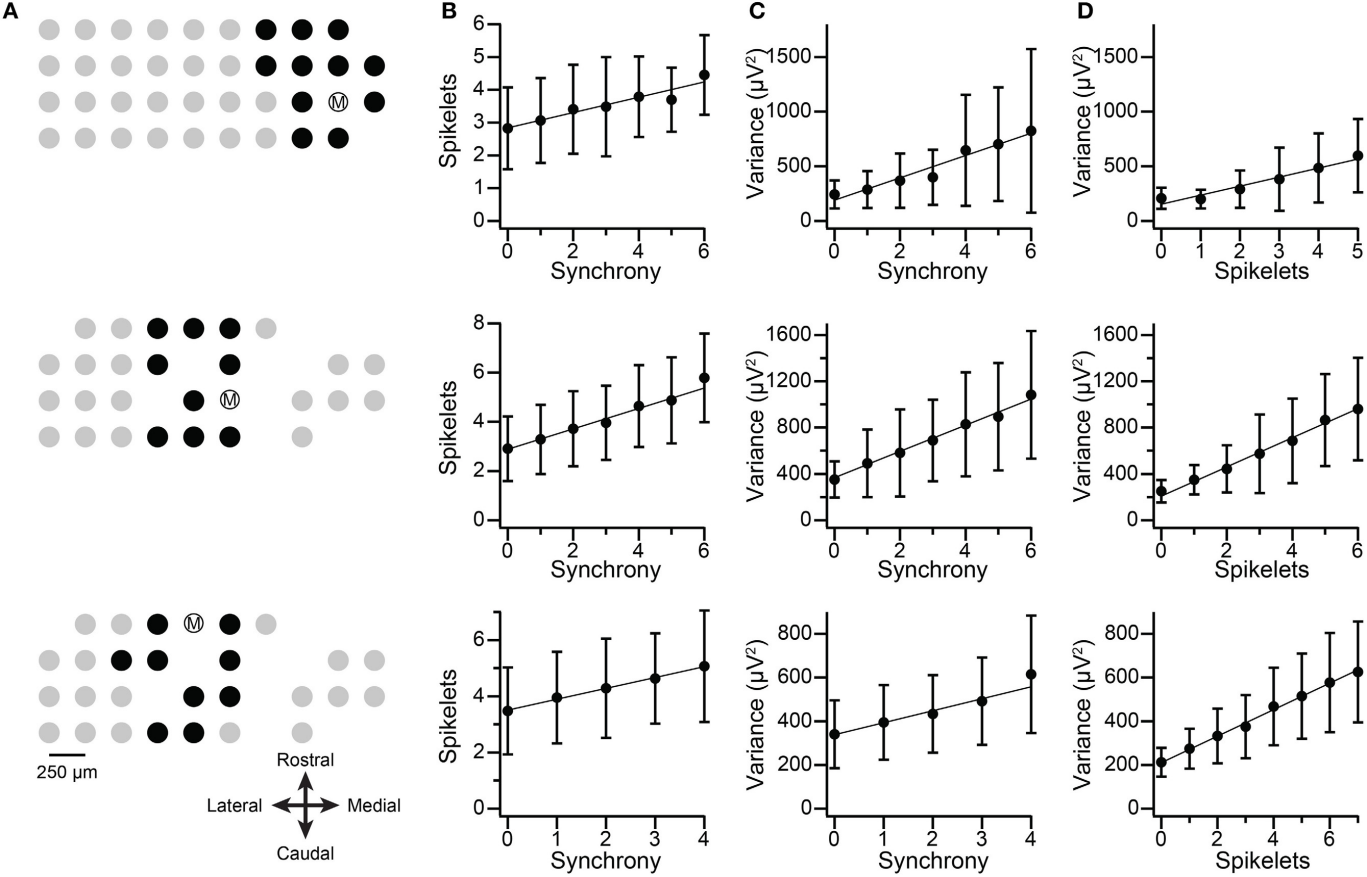
**Synchronous CSs show increased number of spikelets and spikelet-related variance. Each row in figure shows data from one PC. (A)** Recording electrode arrays on crus IIa. Dots show relative positions of electrodes. Encircled M indicates the PC being analyzed. Black dots indicate PCs chosen to be part of the group with cell M for the purpose of the synchrony analysis. **(B)** Plots of the average number of spikelets as a function of synchrony. Synchrony values are the number of PCs in the group that fired synchronously with cell M. **(C)** Plots of the average spikelet-related (T–window) variance as a function of synchrony. **(D)** Plots of spikelet-related variance as a function of spikelet number. In **(B–D)** the data points shown are the mean ±*SD* at each *x*-value. The regression lines are fits to the entire data set for each cell (top, middle, bottom row: *n* = 1135, 1014, and 680 CSs). Middle and bottom row PCs are from the same experiment and had largely overlapping groups.

The spikelets of spontaneous CSs were counted for three cells from two multielectrode experiments (Figure 12, each row shows the data from one cell). For each cell, the level of synchrony, C(0), was calculated for all cell pairs it formed with other PCs in the recording array, and the distribution of synchrony for each cell was examined. The three PCs were chosen because they showed synchronous activity with a defined group of neighboring PCs and had relatively large spikelets. The recording arrays and the cell groups are shown in Figure 12A. The spikelet counts were done blindly with respect to knowledge of the synchronization of the individual CSs. Once the counts were completed, the CSs were sorted according to their level of synchronization with CSs from other PCs in the group. For each cell the correlation between synchrony level among group members and spikelet number was determined, and a regression line was fit to the data (*n* = 1135, 1014, and 680 CSs). The correlation of the spikelet counts to the synchrony level was significant in each case (*r* = 0.23, 0.36, and 0.25; *p* < 1 × 10^−10^). Moreover, plots of the average spikelet number at each level of synchrony (number of other PCs firing synchronously with the reference cell) show a nearly perfect correlation (*r* = 0.97, 0.99, and 0.998), and the regression lines (fit to the entire dataset from each PC) show no systematic deviation from the averages. This suggests a linear relationship between synchrony and spikelet number (Figure 12B), which was confirmed statistically (MSS_LOF_/MSS_PE_ ratio, *p* > 0.05). In sum, these results directly demonstrate that spikelet number increases with synchrony, albeit for a limited sample of PCs.

For comparison, the correlation between the spikelet-related (T-window) variance and the synchrony for these PCs was tested, and were consistent with the results of using spikelet number (*r* = 0.44, 0.45, and 0.32; Figure 12C), although, interestingly, the *r* values were somewhat higher than those for direct spikelet counts. Correlation of average variance with synchrony produced near perfect correlations (0.97, 0.995, and 0.97), and tests for goodness of fit indicated a linear relationship (MSS_LOF_/MSS_PE_ ratio, *p* > 0.05) for two of three PCs. Lastly, we compared spikelet number and variance to each other directly. Once again, significant correlations were found (Figure 12D; all data: *r* = 0.46, 0.58, 0.56; averages: *r* = 0.97, 0.996, 0.998).

## Discussion

The major findings of this paper indicate that a causal relationship exists between CS synchrony and the variance of the recording signal during the spikelet portion of the CS. This variance was shown to reflect the shape and number of spikelets comprising the CS, suggesting that CS synchrony may be a parameter related to the characteristics of the spikelets themselves. Spikelet size and number have been linked to the axonal output of the PC (Ito and Simpson, 1971; Khaliq and Raman, 2005; Monsivais et al., 2005), and to the degree and type of plasticity induced by CS activity (Mathy et al., 2009). Thus, the results raise the possibility that synchrony is an important control parameter for both the motor coordination and motor learning functions that have been proposed for the olivocerebellar system.

The validity of these conclusions, however, rests, in the first place, upon whether the changes in the measured variance with synchrony actually reflect changes in the CS waveform of a PC, as opposed to changes in the electric fields generated by synchronized activity among cells that surround the PC. Thus, we first discuss whether such field effects can fully explain the observed correlation or whether changes in the variance can, at least partly, be ascribed to changes in the CS waveform.

### Is the correlation of spikelet-related variance and CS synchrony due to fields from surrounding cells?

The CS recordings were made with extracellular microelectrodes. Local field potentials, due to the summed activity of simultaneously active nearby cells, may also be recorded along with the single unit activity by such electrodes. However, the contribution of such fields was minimized by using high pass filters with cutoff frequencies in the 300–400 Hz range. Such filtering, in particular, should strongly attenuate fields associated with relatively long lasting events, such as synaptic potentials, which are usually the major sources of fields.

Nevertheless, the geometrical arrangement of the PCs, and the ability of the olivocerebellar system to activate PCs synchronously, provide the potential substrate for substantial fields to be generated by faster events, namely CSs. Indeed, a significant field is produced in the molecular layer following electrical stimulation of the IO, and likely reflects both synaptic and spike activity (Eccles et al., 1966a). While electrical stimuli evoke precisely synchronized CSs in greater numbers of PCs than would normally occur with spontaneous activity, the fields associated with spontaneous CS activity, although smaller in absolute size, should still correlate with the level of synchrony, and thus might lead to a correlation between the variance we measured during the T window and synchrony.

A number of the results (i.e., the baseline and N window variance measurements) address this issue. Overall, they indicate that, although fields were detected by our electrodes along with the single unit activity, and sometimes contributed significantly to the increase in variance measured during the spikelet period, their contribution generally only accounted for at most a small fraction of the observed change in spikelet-related variance (T window) with synchrony level.

The precision of CS synchrony is important for drawing conclusions from these measurements. Specifically, the central peak in cross-correlograms of CS activity between two PCs often has a width of 5–20 ms (Bell and Kawasaki, 1972; Sasaki et al., 1989). Moreover, the CS itself lasts on the order of 10 ms. Thus, if fields from the CS activity of surrounding cells were to have a significant effect on the signal being recorded by a particular electrode, such an effect should be present for a significant time period surrounding the CSs recorded by the electrode. Such an effect was searched for using the N window, but the variance during this window was essentially identical to the general baseline, from which we conclude that fields from the spontaneous CS activity of surrounding PCs do not generally make significant contributions to the variance of the CS signal.

However, it is still possible that during highly synchronous events, detectable effects of the fields on the variance might occur, leading to a correlation. Measurements of the baseline just preceding the CS indicates that this does not usually happen, as a significant correlation between synchrony and the baseline variance was not found in the majority of cells. Even for the remaining PCs, where evidence for a significant effect was detected, comparison of the slope of the regression lines indicated that the increase in variance due to the fields accounted for a relatively small fraction (20–25%) of the total increase that was observed, in most cases. One caveat should be noted. Even when the baseline variance was measured from the 1-ms period just before the CS onset, the level of synchronization might be less, and thus the fields smaller, at that point relative to during the CS itself. However, given the typical width (5–20 ms) of the central peak in the CS cross-correlograms it seems unlikely that the fields would be diminished so significantly in such a short interval. Moreover, the analysis of the fields-only traces also showed a similarly weak correlation with shallow regression line slopes, and in this case the variance was measured during what should be the time when the T window variance is measured when a CS is present.

In sum, these results indicate that although fields from surrounding cells may contribute to the measured signal variance during the spikelet portion of the CS, this contribution cannot fully explain the relationship of the variance with synchrony levels. Thus, we conclude that much of the correlation between the T window variance and synchrony is due to changes in the waveform of the CS itself.

### Is the correlation between spikelet-related variance and CS synchrony due to increased spikelet number or changes in their shape?

The increase in T window variance of synchronous CSs could indicate greater numbers of spikelets and/or a change in the shape of individual spikelets, such as increased amplitude or width (duration); however, actual counts of spikelets in several PCs provide direct evidence that synchronous CSs tend to have larger numbers of CSs. Whether they also show increased amplitudes or duration needs to be determined; however, the variance data can address the question of whether both factors co-vary with synchrony. For the majority of PCs, a linear relationship existed between synchrony and variance, which strongly suggests that spikelet number and shape do not simultaneously co-vary with the synchrony level, because a non-linear relationship would likely result if they did. This is consistent with the linear relationship of T window variance and spikelet number shown by the majority of PCs. For PCs with non-linear relationships between synchrony and variance, no consistent pattern was found, with some curves showing a plateau and others showing a supralinear trend (data not shown). The plateau relationship is consistent with the negative correlation of the S window variance with spikelet number shown by a minority of PCs. The supralinear curves do not correspond to either of the patterns observed when looking at the general relationship between spikelets and variance levels, which suggests that certain types of changes in the spikelet character driven by synchrony may not follow the standard relationships between spikelet number and shape.

### Functional consequences of modulating CS waveform

The olivocerebellar system has been proposed to have roles in both motor coordination (Llinás, 1991) and motor learning (Ito, 2001). For its motor coordination role, the focus is on how the CS activity can alter the axonal output of the PC and how that influences cerebellar nuclear activity. In contrast, for motor learning, the focus is not on the CS's contribution to PC output, but rather on how the CS gates changes in the strength of the parallel fiber-PC synapse. In both cases, the number of spikelets is a potential way for allowing the CS to make a contribution that is distinguishable from that from simple spikes. Indeed, the type of plasticity induced, LTP or LTD, by a CS has been related to its number of spikelets (Mathy et al., 2009). In contrast, for affecting nuclear cell activity, the direct effect of synchronous activity among PCs that converge on the same cell is the usual mechanism thought to be involved, and a number of results are consistent with this interpretation (Llinás and Mühlethaler, 1988; Bengtsson et al., 2011; Blenkinsop and Lang, 2011; Lang and Blenkinsop, 2011). An increase of spikelets with synchrony (as the present suggest should happen) would amplify the effect of convergence, and further help distinguish CS signals from the tonic simple spike activity, because at least some spikelets are transmitted down the PC axon at short intervals (Ito and Simpson, 1971; Khaliq and Raman, 2005; Monsivais et al., 2005).

Finally, we recently proposed that the olivocerebellar system may participate in both motor control and motor learning processes (Schweighofer et al., 2013). Having a dual function raises the issue of whether and how the olivocerebellar system can selectively act in one capacity, as it would seem problematic to initiate significant changes in connectivity with every motor command and vice versa. Thus, it was further proposed that synchrony level could act as a switching mechanism, such that at lower synchrony levels CSs would gate plasticity but would not cause significant changes in ongoing cerebellar output, and that highly synchronized activity would affect ongoing cerebellar output directly (Schweighofer et al., 2013). The increase in spikelets with high synchrony described here could be a mechanism to further enhance the difference between high and low levels of synchronous CS activity, and thereby help limit motor commands to the high synchrony realm.

### Conflict of interest statement

The authors declare that the research was conducted in the absence of any commercial or financial relationships that could be construed as a potential conflict of interest.
